# Atrial Myocyte Function and Ca^2+^ Handling Is Associated with Inborn Aerobic Capacity

**DOI:** 10.1371/journal.pone.0076568

**Published:** 2013-10-16

**Authors:** Anne Berit Johnsen, Natale P. L. Rolim, Tomas Stølen, Marcia Alves, Mirta M. L. Sousa, Geir Slupphaug, Steven L. Britton, Lauren G. Koch, Godfrey L. Smith, Ulrik Wisløff, Morten A. Høydal

**Affiliations:** 1 K.G. Jebsen Center of Exercise in Medicine, Department of Circulation and Medical Imaging, Faculty of Medicine, Norwegian University of Science and Technology, Trondheim, Norway; 2 Department of Cancer Research and Molecular Medicine, Norwegian University of Science and Technology, Trondheim, Norway; 3 The Proteomics and Metabolomics Core Facility, Norwegian University of Science and Technology and the Central Norway Regional Health Authority, Trondheim, Norway; 4 Department of Physical Medicine and Rehabilitation, University of Michigan, Ann Arbor, Michigan, United States of America; 5 Institute of Biomedical and Life Sciences, University of Glasgow, Glasgow, United Kingdom; Loyola University Chicago, United States of America

## Abstract

**Background:**

Although high aerobic capacity is associated with effective cardiac function, the effect of aerobic capacity on atrial function, especially in terms of cellular mechanisms, is not known. We aimed to investigate whether rats with low inborn maximal oxygen uptake (VO_2 max_) had impaired atrial myocyte contractile function when compared to rats with high inborn VO_2 max_.

**Methods and Results:**

Atrial myocyte function was depressed in Low Capacity Runners (LCR) relative to High Capacity Runners (HCR) which was associated with impaired Ca^2+^ handling. Fractional shortening was 52% lower at 2 Hz and 60% lower at 5 Hz stimulation while time to 50% relengthening was 43% prolonged and 55% prolonged, respectively. Differences in Ca^2+^ amplitude and diastolic Ca^2+^ level were observed at 5 Hz stimulation where Ca^2+^ amplitude was 70% lower and diastolic Ca^2+^ level was 11% higher in LCR rats. Prolonged time to 50% Ca^2+^ decay was associated with reduced sarcoplasmic reticulum (SR) Ca^2+^ ATPase function in LCR (39%). Na^+^/Ca^2+^ exchanger activity was comparable between the groups. Diastolic SR Ca^2+^ leak was increased by 109%. This could be partly explained by increased ryanodine receptors phosphorylation at the Ca^2+^-calmodulin-dependent protein kinase-II specific Ser-2814 site in LCR rats. T-tubules were present in 68% of HCR cells whereas only 33% LCR cells had these structures. In HCR, the significantly higher numbers of cells with T-tubules were combined with greater numbers of myocytes where Ca^2+^ release in the cell occurred simultaneously in central and peripheral regions, giving rise to faster and more spatial homogenous Ca^2+^-signal onset.

**Conclusion:**

This data demonstrates that contrasting for low or high aerobic capacity leads to diverse functional and structural remodelling of atrial myocytes, with impaired contractile function in LCR compared to HCR rats.

## Introduction

Despite the important contribution of atria to refilling of the ventricles during increased workload such as physical activity, there are limited data on the association between atrial function and aerobic capacity, especially in terms of cellular mechanisms. Recent studies have shown that failure to increase atrial function on exercise impairs compensatory late diastolic filling with increased heart rate. This contributes to genesis of exertional dyspnoea [1], [2]. It is well established that aerobic exercise training improves left ventricular cardiac function with increased cardiac output during systole and faster relaxation during diastole [3]. These functional alterations of the heart are also supported by several studies which indicate a clear association between both training induced and high inborn aerobic capacity with improved left ventricular myocyte function and Ca^2+^ handling (reviewed in Kemi *et al.* [4]). How inborn aerobic capacity influences on atrial myocyte function and Ca^2+^ handling is presently not known.

Here we apply a model of rats with diverging inborn aerobic running capacities [5] to study the association between intrinsic aerobic fitness and atrial myocyte function and Ca^2+^ handling. Rats with low inborn aerobic running capacity (Low Capacity Runners; LCR rats) have a high-risk cardiovascular profile whereas rats with high inborn aerobic running capacity (High Capacity Runners; HCR rats) developes a healthy athletic profile with improved cardiac function [6]. We hypothesised that LCR rats have impaired atrial myocyte function associated with defective intracellular Ca^2+^ handling compared to HCR rats.

## Methods

### Animal Model

LCR and HCR rats were artificially selected and bred over 22 generations on the basis of difference in inborn running capacities between two populations, the LCR and HCR rats. Breeding started from N:NIH stock obtained from the National Institute of Health (USA), as described previously [5], [6].

The Norwegian Council for Animal Research approved the study, which was in accordance with the Guide for the Care and Use of Laboratory Animals by the European Commission Directive 86/609/EEC.

### Maximal Oxygen Uptake (VO_2 max_) Measurement

VO_2 max_ was measured by uphill (25°) treadmill running in a metabolic chamber until exhaustion as previously described [7], [8].

### Atrial Myocyte Isolation

Left atria from rats were isolated using a modified mouse model protocol [9]. After removal, hearts were kept in ice-cold cell isolation buffer (130 mM NaCl, 5.4 mM KCL, 0.5 mM MgCl_2_, 0.33 mM NaH_2_PO_4_, 22 mM glucose, 50 µU/ml bovine insulin (I-5500, Sigma), 25 mM HEPESNaOH (pH = 7.4)) with 0.4 mM EGTA and immediately canulated through aorta and retrogradely perfused (7.5 ml/min, 37 C) with isolation buffer containing 0.4 mM EGTA for 2–4 min. Then the hearts were perfused with the enzyme solution containing isolation buffer supplemented with 0.048 mM CaCl_2_ and 1 mg/ml collagenase (Type II, Worthington, 295 U/mg). From the digested hearts (10–15 minutes perfusion) left atria were removed, cut into 3–5 pieces, and further digested by gentle stirring for 5–10 min in fresh enzyme solution until myocytes appeared. Tissue chunks were then transferred to isolation buffer containing 0.096 mM CaCl_2_ and 10 mg/ml 0.1% bovine serum albumin (Sigma), cut into as small pieces as possible and mechanically agitated with a pipette. The cell suspension was centrifuged at 40×g for 2 minutes in a 15 mL plastic centrifuge tube, the supernatant was gently removed and the cell pellet was resuspended in 2 ml of isolation buffer with 0.026 mM CaCl_2_.

### Ca^2+^ Measurements

For intracellular Ca^2+^ recordings, Ca^2+^ concentration in the perfusion buffer was increased to 1.8 mM. Fura-2/AM-loaded (20 minutes in 2 µM, Molecular Probes, Eugene, OR) cardiomyocytes were field stimulated by bipolar electrical pulses at 2 Hz and then 5 Hz on an inverted epifluorescence microscope (Nikon TE-2000E, Tokyo). Cell shortening was measured by video-based sarcomere spacing (Ionoptix, Milton, MA) and intracellular Ca^2+^ concentration was measured by counting 510 nm emissions with a photomultiplier tube (PMTACQ, IonOptix, Milton, MA) after exciting with alternating 340 and 380 nm wavelengths (*F^340/380^* ratio) (Optoscan, Cairn Research, Kent, UK). Quantification of the Sarcoplasmic reticulum (SR) Ca^2+^ content and rate constant for fractional contribution of Ca^2+^ removal by SR Ca^2+^ ATPase (SERCA2a) and Na^+^/Ca^2+^ exchanger (NCX) are previously described in Seidler *et al*. [10].

A method similar to that established by Shannon *et al*. [11] was used to determine diastolic Ca^2+^- leak from the SR. To bring the cellular Ca^2+^-content to a steady state, cardiomyocytes were electrically stimulated at 1 Hz in normal HEPES based 1.8 mM Ca^2+^-solution for 30–60 seconds. After the last electric stimulus, perfusion was switched to a 0 Na^+^/0 Ca^2+^ containing solution and diastolic Ca^2+^ concentration was measured in quiescent non-stimulated cardiomyocytes (40 seconds) ± Tetracaine (1 mmol/L). The 0 Na^+^/0 Ca^2+^ solution prevents the Na^+^ - Ca^2+^ exchange, which is the primary Ca^2+^-influx and efflux mechanism at rest. Tetracaine blocks the Ca^2+^-leak over the RyR. The quantitative difference between diastolic Ca^2+^-concentration with and without tetracaine determines leak. After the 40 second period in 0 Na^+^/0 Ca^2+^ ± Tetracaine solution, caffeine was added (10 mM) to assess the SR Ca^2+^-content. Diastolic Ca^2+^-leak is presented as diastolic [Ca^2+^]_i_ in relation to total SR Ca^2+^-content.

### Confocal Microscopy

Imaging of T-tubular network and spatiotemporal characteristics of Ca^2+^ transients were studied using a laser scanning microscope (LSM 5 PASCAL, Zeiss, Jena, Germany) and a Zeiss 63×1.23NA oil-immersion objective. To visualize T-tubular network, quiescent, non-perfused cardiomyocytes loaded with the membrane specific Di-8-ANEPPS dye (10 µM, Molecular Probes) were confocal Z-stack scanned (488 nm excitation and detection at >514 nm). This was performed with pinhole of 1 airy unit and 0.38 micron thick stacks. T-tubule density was analyzed with custom-made applications in IDL 6.0 (ITT Visual, Boulder, CO, USA), by counting pixels stained with the dye relative to the total number of pixels after removing pixels associated with the external cell membrane. To study spatiotemporal characteristics of Ca^2+^ transients, Fluo-3/AM (10 µM, Molecular Probes) loaded cardiomyocytes were confocal line-scan recorded (488 nm excitation and detection at >514 nm) during steady state stimulation at 1 Hz. Repetitive scanning of a line parallel to the transversal axis of the cell were used to visualize Ca^2+^ signal. For the Ca^2+^ synchrony analysis, the transients were divided into 5 equal strips. Time from stimulation to 50% peak Ca^2+^ release was measured for each strip by the programme LabTalk Origin (OriginLab Corporation, Northhampton, MA) to determine spatial differences in systolic rise time of the Ca^2+^ transient from the edges to the center of the cardiomyocytes.

### Western Blot Analyses

Proteins (100 µg total lysate) from left atrium were heated in LDS loading buffer (Invitrogen) and subjected to electrophoresis on pre-cast 3–8% Tris-acetate denaturing NuPAGE gels (Invitrogen). After separation for 3 hours at 150 V/220 mA and 4°C, gels were incubated in 2× NuPAGE transfer buffer (Invitrogen) contatining 0.02% SDS for 10 minutes. And proteins were electro-transferred onto PVDF membranes (Immobilon-FL, Millipore) at 20 V overnight and 4°C (BioRad, Hercules, CA). The membranes were blocked with Odyssey blocking buffer (LiCOR) prior to incubation with monoclonal anti-ryanodine receptor (RyR2) (1∶5,000; Thermo Fisher Scientific, Waltham, MA), polyclonal anti-pS2809-RyR2 (1∶1,000; Badrilla, Leeds, UK), and monoclonal anti- glyceraldehyde 3-phosphate dehydrogenase (GAPDH) (1∶100,000; Millipore (Chemicon), Temecula, CA) antibodies overnight at 4°C. After incubation with secondary goat anti-mouse IRDye800LT and goat anti-rabbit IRDye680LT secondary antibodies (Li-COR) bands were detected using an Odyssey infrared imaging system (Li-COR, Lincoln, NE). Quantitative analyses were performed with Odyssey v.3.0 software and ImageJ Data Acquisition Software (National Institute of Health, Bethesda, MD).

### Statistics

Data are presented as mean ± SD. Student T-test was used to identify statistical differences between the groups. Man-whitney Rank Sum test was used if normality test (Shapiro-Wilk) failed. The Fisher’s Exact test was applied to the categorical data. P<0.05 was considered statistical significant.

## Results

### Intrinsic Aerobic Capacity and Cardiac Contractility

**Figure 1:**
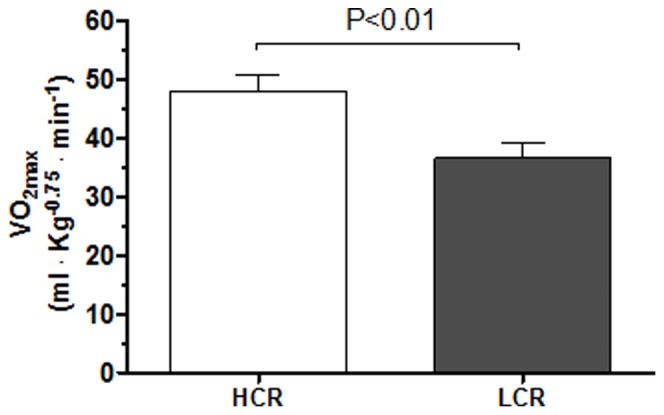
Aerobic capacity measured by maximal oxygen uptake (VO_2 max_) in Low Capacity Runner (LCR) and High Capacity Runner (HCR) rats. Data are presented as mean±SD.

VO_2 max_ was 24% lower in LCR rats compared to HCR rats (Figure 1, p<0.01).

### Atrial Myocyte Function

**Figure 2:**
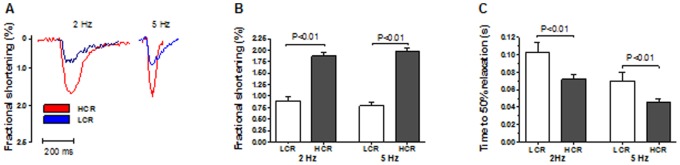
Analysis of atrial myocyte function. A, Exemplary tracings of atrial myocyte function in Low Capacity Runner (LCR)- compared to High Capacity Runner (HCR) rats display a deteriorated viability in LCR rats both at systolic and diastolic properties. B, Fractional shortening was depressed at 2 and 5 Hz stimulation in LCR rats and, C, Time to 50% relaxation was increased LCR rats. n = 5 animals, n = 4−6 cells from each animal. Data are presented as mean±SD.

Fractional shortening in atrial myocytes from LCR was 52% lower at 2 Hz and 60% lower at 5 Hz stimulation (Figure 2B, p<0.01) compared to that observed in HCR. Diastolic atrial myocyte function, measured as time to 50% re-lengthening was 43% (2 Hz) and 55% (5 Hz) slower in LCR rats (Figure 2C, p<0.01).

### Ca^2+^-handling

**Figure 3:**
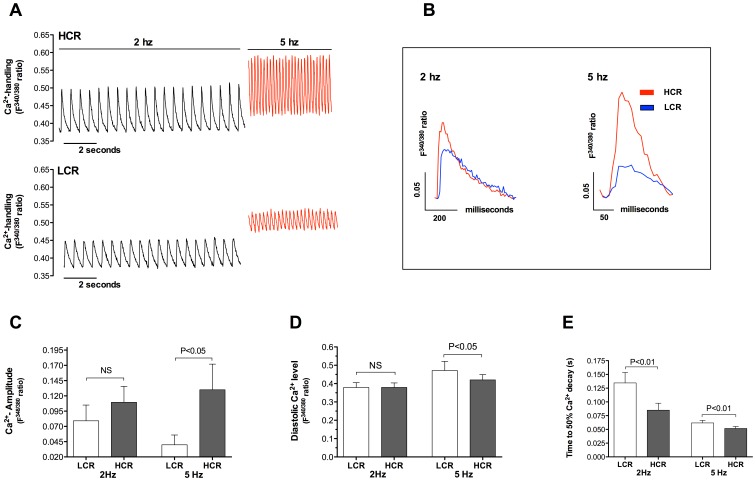
Ca^2+^-handling properties determined in isolated FURA2/AM loading atrial myocytes during increasing stimulation frequency from 2–5 Hz. A, Exemplary recordings of Ca^2+^-transients in Low Capacity Runner (LCR)- and High Capacity Runner (HCR) rats. B, Exemplary tracings of one single twitch Ca^2+^ transient at 2 hz (left panel) 5 hz (right panel) with comparison of LCR and HCR (normalized diastolic Ca^2+^ levels). C, Ca^2+^-amplitude during systolic contraction of the atrial myocytes. D, Diastolic Ca^2+^-level measured at end diastole. E, Time to 50% Ca^2+^-removal during diastole. All Ca^2+^-recordings are presented as the 340/380 ratio of FURA2/AM. n = 5 animals, n = 4−6 cells from each animal. Data are presented as mean±SD.

We found that atrial myocyte Ca^2+^- handling was significantly impaired in LCR rats compared to HCR rats. Exemplary tracings of Ca^2+^ transients are shown in figure 3A and 3B. At 2 Hz stimulation the Ca^2+^-amplitude was similar in both groups, whereas it was 70% lower in atrial myocytes from LCR rats at 5 Hz (Figure 3C, p<0.05). Diastolic Ca^2+^-levels were also similar between groups when studied at 2 Hz stimulation but significantly elevated in LCR rats at 5 Hz (Figure 3D, p<0.05). In line with the prolonged time to cell relengthening in atrial myocytes from LCR rats, time to 50% Ca^2+^- decay was significantly longer at both 2 and 5 Hz stimulation when compared to that observed in HCR (Figure 3E, p<0.01).

### Sarcolemmal and SR Ca^2+^-cycling

**Figure 4:**
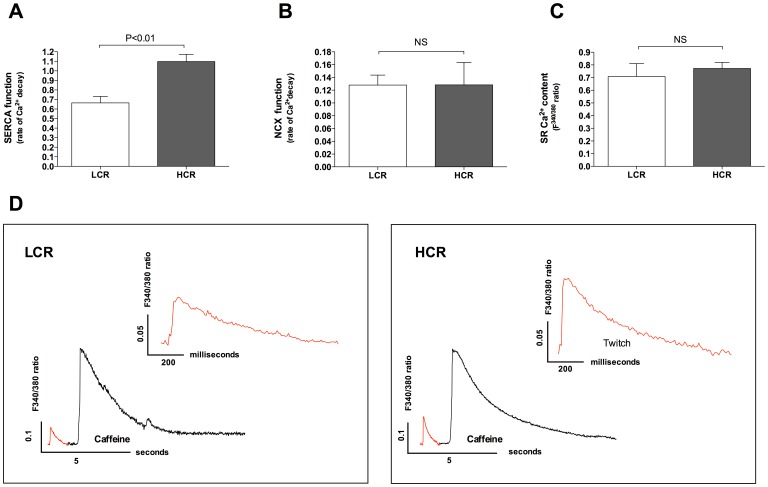
Measurements of sarcoplasmic reticulum (SR) and sarcolemmal Ca^2+^-handling properties. Total SR Ca^2+^ content was measured by assessing peak Ca^2+^ amplitude after rapidly applying Caffeine (10 mM) to the perfusion solution immediately after stopping the electrical stimulation in normal HEPES solution. To quantify the SERCA2a function, a simple model was used based on the following assumptions: SERCA2a transport rate is: K_twitch_ – K_Caffeine/NCX_, where K_twitch_ is the Ca^2+^ removal (F^340/380^ ratio) during the time period from peak electrical stimulated twitch Ca^2+^ to 50% Ca^2+^ decay in normal HEPES solution and the K_Caffeine/NCX_ is the Ca^2+^ removal (F^340/380^ ratio) during the time period from peak caffeine induced Ca^2+^ release to 50% of decay (10 mM Caffeine+HEPES). In presence of caffeine the SERCA is inhibited and the Ca^2+^ removal in this condition is mainly determined by NCX. A, SR Ca^2+^ ATPase (SERCA2a) function was significantly lower in Low Capacity (LCR) rats than High Capacity Runner (HCR) rats. B, Na^+^/Ca^2+^ exchanger (NCX) function was not different between groups. C, SR Ca^2+^-content assessed by application of 10 mM of caffeine after electrical 1 Hz stimulation did not reveal any difference LCR and HCR atrial myocytes. n = 5 animals, n = 4−6 cells from each animal. Data are presented as mean±SD. D, Exemplary recordings of twitch Ca^2+^ transients (red lines) compared to Caffeine transients (black lines). Twitch Ca^2+^ transients are magnified in respective figures for better evaluation of Ca^2+^ handling kinetics.

**Figure 5:**
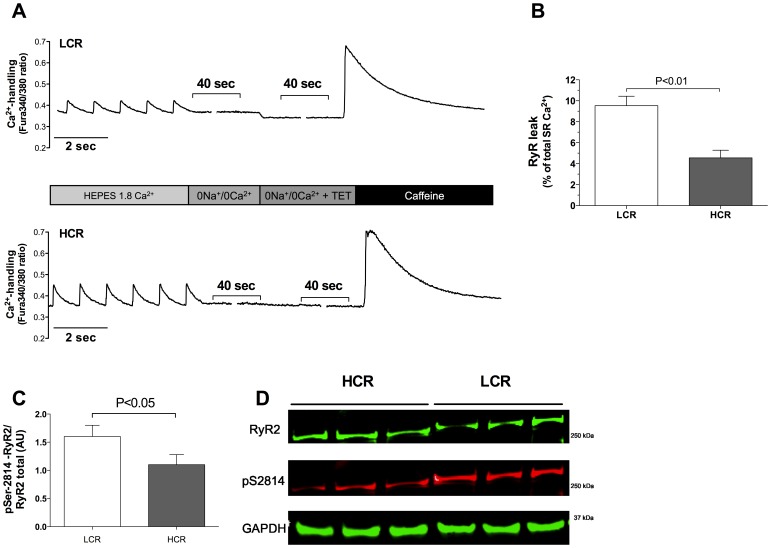
Recordings of diastolic sarcoplasmic reticulum (SR) Ca^2+^ leak after 1 Hz electrical stimulation in normal HEPES 1.8 mM Ca^2+^ solution. A, Exemplary recordings show the protocol of quantification of SR Ca^2+^-leak by determination of diastolic Ca^2+^-levels in quiescent atrial cells with 0 Na^+^/0 Ca^2+^ in the external perfusion solution compared to perfusion solution with 0 Na^+^/0 Ca^2+^+Tetracaine (TET) that inhibits the opening of the ryanodine receptor (RyR2). Recordings were followed by Caffeine (10 mM) induced Ca^2+^ depletion of the SR to determine SR Ca^2+^ storage B, Diastolic SR Ca^2+^-leak was significantly increased in Low Capacity Runner (LCR) rats compared to High Capacity Runner (HCR) rats. n = 5 animals, n = 4−6 cells from each animal. C, Western blot analyses of the ratio between phosphorylated Serine-2814/RyR2 display a significant higher expression in LCR rats (n = 4) compared to HCR rats (n = 3). D, Representative Western blots. Data are presented as mean±SD.

Prolonged time to 50% Ca^2+^-decay was associated with a 39% reduction in Ca^2+^-removal via SERCA2a in atrial myocytes from LCR rats when compared to HCR (Figure 4A, p<0.01). NCX activity was comparable between the groups (Figure 4B). SR Ca^2+^-content was not different between LCR and HCR rats (Figure 4C). Measuring Ca^2+^ in quiescent cardiomyocytes over a prolonged period of time (1 min) with and without tetracaine provides a quantitative assessment of SR (RyR2) Ca^2+^ leak (Figure 5A). We found that diastolic SR Ca^2+^ leak over the RyR2 was increased by 109% in LCR compared to HCR (Figure 5B). To analyse mechanisms of increased diastolic SR Ca^2+^ leak, RyR2 expression and phosphorylation were quantified. We found that RyR2 phosphorylation at the Ca^2+^-calmodulin-dependent protein kinase-II (CaMKII) specific Ser-2814 site is apparently induced in LCR rats (Figure 5C and 5D). The protein kinase A (PKA) phosphorylation site Serine-2808 was not significantly altered (data not shown).

### Transverse (T)- tubule and Cell Dimensions

**Figure 6:**
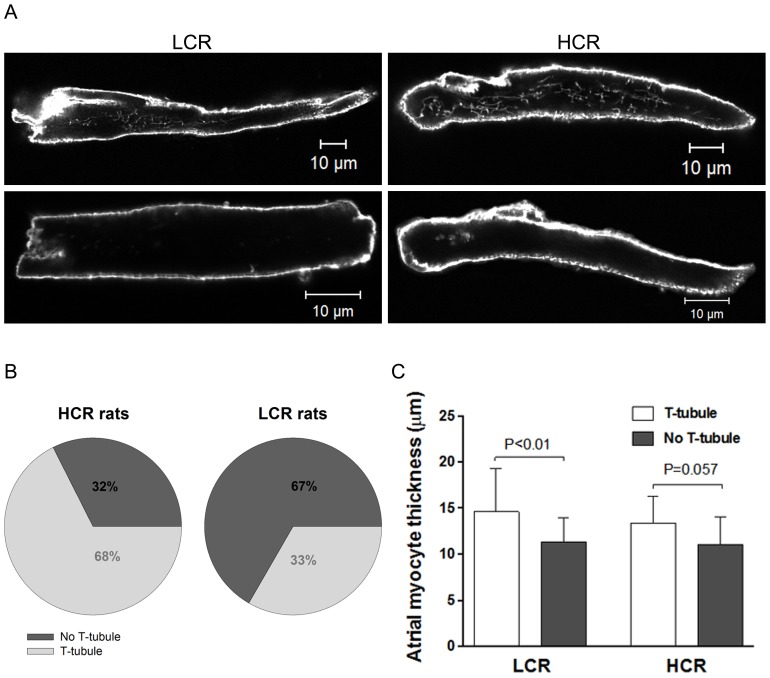
Membrane structures in isolated atrial myocytes. A, Confocal images of Di-8-Anepps stained atrial myocytes with and without T-tubules for Low Capacity Runner (LCR) and High Capacity Runner (HCR) rats. B, Proportion of cells with and without T-tubules for LCR and HCR rats. Absence of T-tubules in the majority of LCR rats may impair Ca^2+^ handling. Comparison of cell thickness in cells with and without T-tubules. Data are presented as mean±SD. n = 57 cells for LCR and 37 cells for HCR.

Synchronous activation of Ca^2+^ -induced Ca^2+^ release is facilitated by T-tubules that are inward invaginations in the plasma membrane that ensure close proximity of L-type Ca^2+^ channels and RyRs in the cell interior We determined T-tubule structure in atrial cells stained with the membrane specific dye Di-8-ANNEPS (typical examples in Figure 6A). We found that fewer atrial cells from LCR had T-tubule structures compared with that observed in HCR (33% in LCR (n = 57 cells) versus 68% in HCR rats (n = 37 cells), P<0.01). However, there was no difference in T-tubule density between the two groups in cells presenting T-tubule structure. In agreement with previous studies from larger animals [12], [13], we observed that isolated myocytes with T-tubules was significantly wider than myocytes without T-tubules (Figure 6B).

### Spatiotemporal Properties of Ca^2+^ Transients

**Figure 7:**
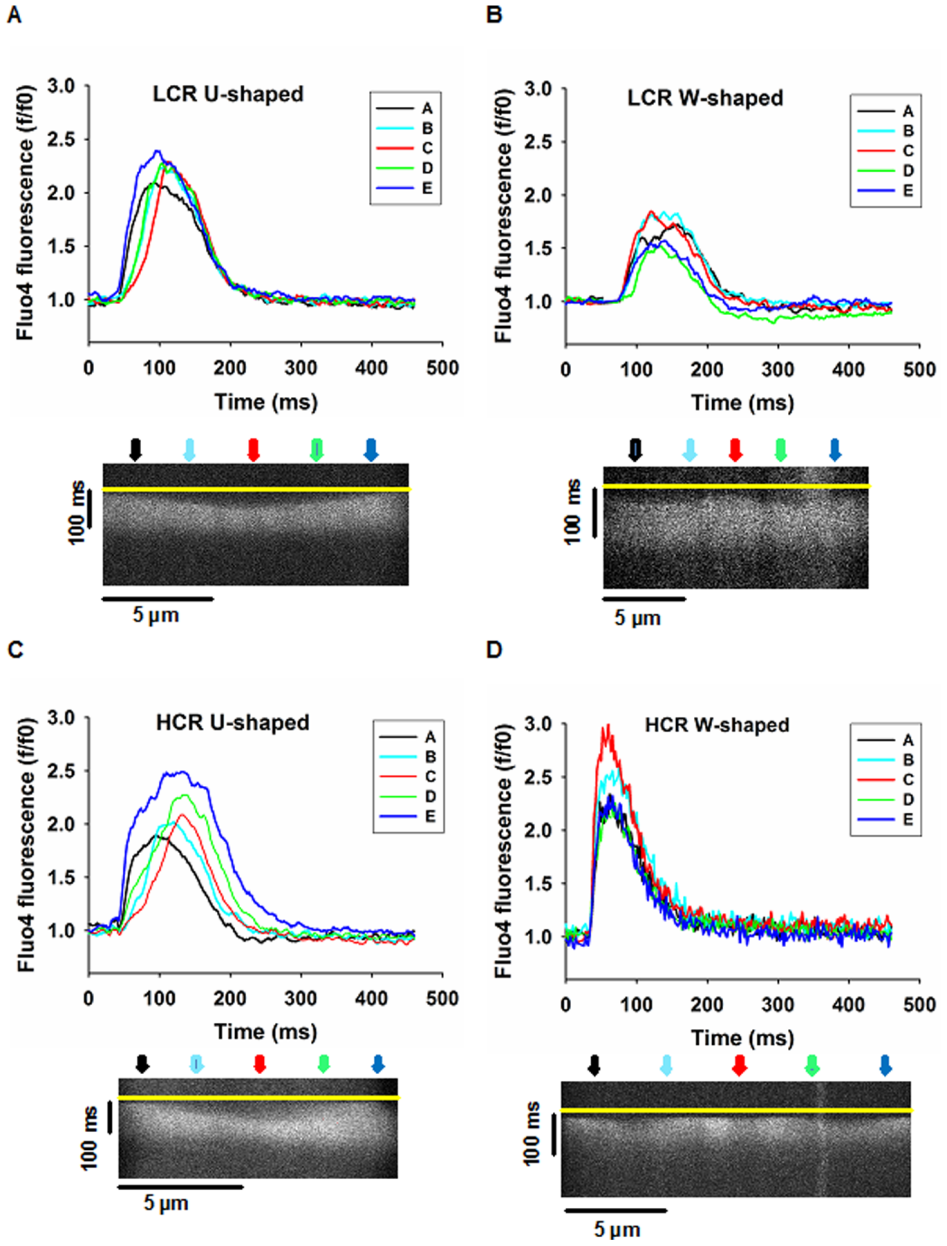
Spatiotemporal characteristics of Ca^2+^ transients in isolated atrial myocytes. Cells were labeled with fluo-4 and confocal line scanned transversely. Panels A–D depict the spatiotemporal properties of Ca^2+^ transient in: A, atrial myocyte with U-shaped Ca^2+^ signal in in Low Capacity Runner (LCR); B, atrial myocyte with W-shaped Ca^2+^ signal in LCR; C, atrial myocyte with U-shaped Ca^2+^ signal in High Capacity Runner (HCR); D, atrial myocyte with W-shaped Ca^2+^ signal in HCR.

**Figure 8:**
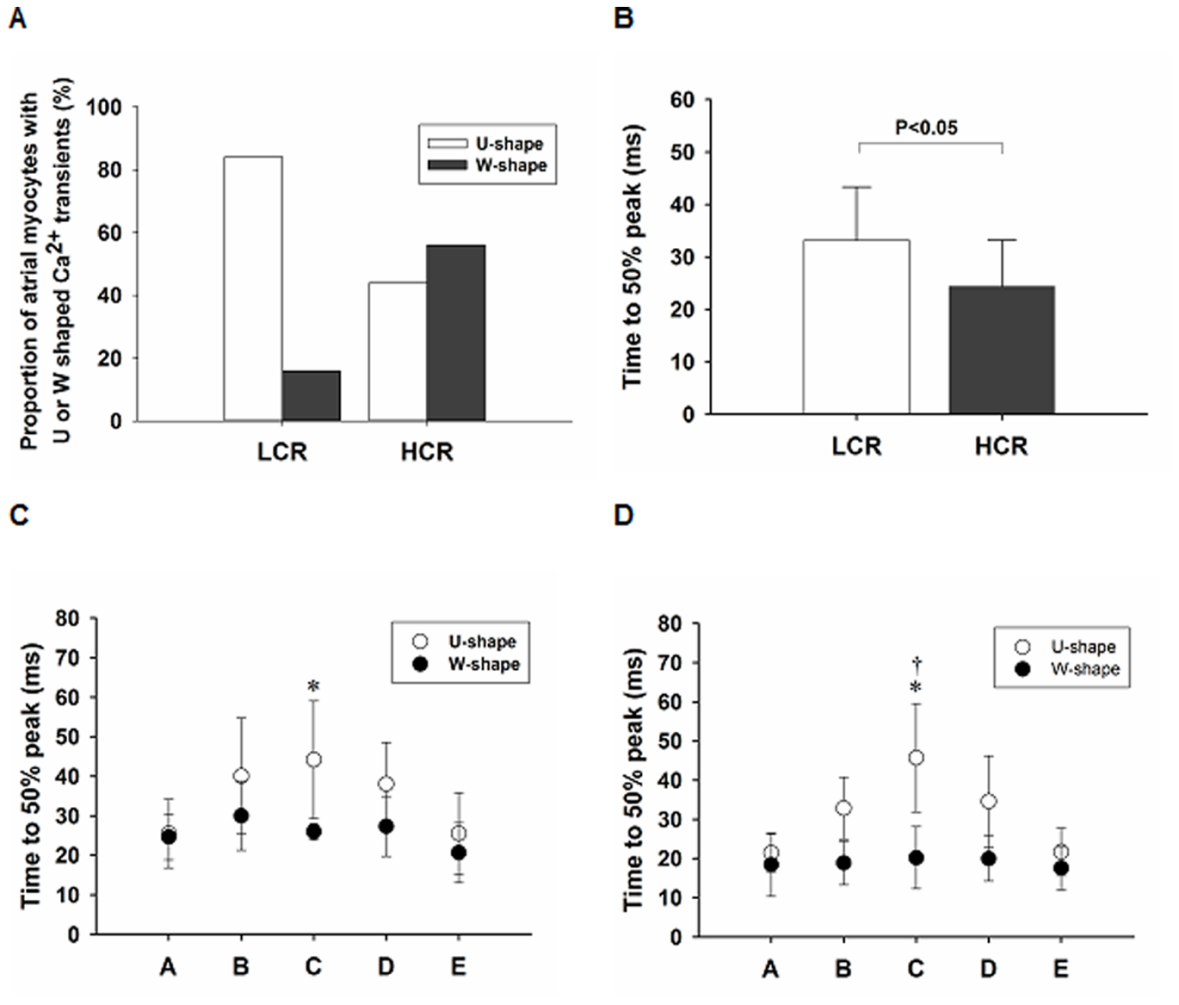
Analysis of transverse linescan Ca^2+^ signal in isolated atrial myocytes. A, Proportion of cells with different Ca^2+^ response pattern (U- or W-shaped). B, Time to 50% peak Ca^2+^ release in Low Capacity Runner (LCR) vs. High Capacity Runner (HCR) rats. C and D, Spatial characteristics of time to 50% peak Ca^2+^ release in U- vs W shaped transients in LCR and HCR. Data are mean±SD. Difference in time to 50% peak Ca^2+^ release between edges (A and E, x-axis) and center (C, x-axis) in U shaped transient: *p<0.05. Difference in time to 50% peak Ca^2+^ release between central region of U- and W-shaped transient: **^†^** *p*<0.05. Data are presented as mean±SD. n = 19 cells for LCR and 16 cells for HCR.

Two types of Ca^2+^ transients were observed in atrial myocytes from LCR and HCR, U-shaped and W-shaped (Exemplary tracings are illustrated in Figure 7), as observed in atrial myocytes in previous rat models [12], [13]. The majority of atrial myocytes from LCR displayed mainly an U- shaped Ca^2+^ transient (84%, n = 19 cells, Figure 8A), where the Ca^2+^ release initiated at the edges of the cells and then propagated inwards. Such response has been observed in cells devoid of T-tubules [12] and is in line with our finding of low proportion of myocytes with T-tubules in LCR. In contrast, the majority of atrial myocytes from HCR displayed W-shaped Ca^2+^ transients (56%, n = 16 cells Figure 8A), where the Ca^2+^ signal initiated at the edges of the cells as well as in the central regions of the cells, giving rise to more complex pattern of transient. LCR had a significant lower proportion of W shaped Ca^2+^ transients compared to HCR and we observed that time to 50% peak Ca^2+^ was slower in LCR than HCR (p<0.05, Figure 8B). Analysis of time to 50% peak of Ca^2+^ transient in U- compared to W-shaped transients revealed that U-shaped transients were slower than W-shaped (p<0.05, from HCR group) and no differences were observed when comparing U- vs. U –shaped transients and W- vs. W shaped transients between groups. This suggests that the slower time to peak in LCR was partly due to high proportion slow U-shaped transients. Further spatiotemporal analysis of U-shaped Ca^2+^ transient revealed that the central Ca^2+^ release within the myocytes was significantly slower than the edges (p<0.05, within LCR and HCR group, Figure 8C and 8D). Furthermore, central Ca^2+^ release in U-shaped Ca^2+^ transients was significantly slower than the corresponding central Ca^2+^ release in W-shaped transients (p<0.01, from HCR group).

## Discussion

This is the first study to demonstrate that low inborn aerobic capacity is directly associated with reduced contractile function and impaired Ca^2+^ handling in atrial myocytes.

### Cardiomyocyte Function and Ca^2+^ Handling

We have previously reported that left ventricular myocytes from LCR rats have impaired systolic and diastolic function relative to HCR rats [6]. Ventricular contractile dysfunction has been strongly associated with altered Ca^2+^ handling in heart failure [14] and such association has also been reported in atrial myocytes in HF model [15]. This study revealed reduced fractional shortening and prolonged time to diastolic re-lengthening combined with depressed atrial myocyte Ca^2+^ handling in LCR compared to HCR rats, which confirms that there is an association between aerobic capacity and development of atrial myocyte function. Ca^2+^ amplitude together with duration of Ca^2+^ transient are main determinants of cardiac contraction [16]. In this study atrial myocyte Ca^2+^ amplitude was preserved at 2 Hz in LCR compared to HCR rats, still fractional shortening was depressed in LCR rats, indicating reduced Ca^2+^ sensitivity. At 5 Hz stimulation there was a significant decrease in Ca^2+^ amplitude in LCR rats. The observed negative frequency dependent alteration in systolic Ca^2+^ amplitude in the LCR (illustrated in Figure 3) is important and likely contributes to limited aerobic capacity during increasing workload such as endurance exercise. In our data there are two mechanisms that potentially may cause this negative response in LCR: 1) reduced reuptake of Ca^2+^ to the SR by SERCA2a and 2) less developed T-tubule structures and reduced initiation sites for Ca^2+^ activated Ca^2+^ release.

Earlier studies have shown that reduced SERCA2a function is related to a negative frequency dependent acceleration of Ca^2+^ removal [17]. When increasing the frequency from 2 Hz to 5 Hz SERCA2a may not have the capacity to cope with the increased demand of rapidly circulating Ca^2+^ and thereby not able to reload the SR with Ca^2+^ available between stimulation. Despite this obvious explanation we were unable to detect any significant difference SR Ca^2+^ content after caffeine-stimulated depletion. The stimulation frequency before caffeine stimulation in our experiments was, however, performed after 1 Hz electrical stimulation, which probably is too low to tax the capacity of SERCA2a. Therefore, despite that the SERCA2a capacity is reduced in LCR already at low frequencies compared to HCR, the capacity may still be adequate to maintain a preserved end-diastolic Ca^2+^ and SR Ca^2+^content at this frequency. Our finding of a significantly increased end-diastolic Ca^2+^ level at 5 Hz stimulation supports a failure of SERCA2a for reuptake of Ca^2+^ during increased Ca^2+^ cycling rates which potentially also mediated a reduced SR Ca^2+^ available for release.

T-tubule system of variable extent has been reported in rat atrial cells [12], [13]. Here we show a greater proportion of cells devoid of any T-tubule system in LCR compared to HCR rats and we suggest that differences in this could be associated with intrinsic aerobic capacity. The high number of U-shaped Ca^2+^ transients in the myocytes from LCR compared to HCR rats, together with relative low number of atrial myocytes with T-tubules in LCR rats, suggests a lack of central initiation sites for Ca^2+^ response. The transients showing this spatial profile rises rapidly at the edges of the myocytes and more slowly in the interior, which is in agreement with association between lack of T-tubules and spatiotemporal characteristics of Ca^2+^ transients demonstrated in atrial cells previously [12], [13], [18]. In cells devoid of T-tubules, the close apposition of L-type Ca^2+^ channels (LTCCs) and RyRs that is necessary for Ca^2+^ induced Ca^2+^ release, occurs only at the cells periphery leading to dyssynchronous Ca^2+^ release [19]. Similar Ca^2+^ dynamics has been reported in ventricular myocytes of HF models because of a loss of or reorganization of T-tubules leaving some orphaned RyRs that become physically separated from LTCCs [20], [21]. The average signal of Ca^2+^ release across the entire spatial dimension of the line scan was faster in HCR rats compared to LCR rats. This may be explained by the relative higher number of W-shaped Ca^2+^ transients due to more developed T-tubular network in HCR myocytes, which provide central initiation sites for Ca^2+^ release with faster and more spatial homogenous onset of Ca^2+^-signal. This is supported by Smyrnias *et al*. [13] who found cells with W-shaped Ca^2+^ transients to have significantly faster recovery of systolic Ca^2+^ amplitude after complete depletion of Ca^2+^ by caffeine application. At increasing frequencies the functional consequences of delayed central Ca^2+^ rise in LCR rats will be even more pronounced because of the increased demand of rapid initiation of Ca^2+^ induced Ca^2+^ release. Therefore, we suggest an association between the observed differences in spatio-temporal characteristics of Ca^2+^-signal and the observed differences in atrial myocyte systolic performance due to the fact that slow rise in Ca^2+^ release may limit synchronous contractile activation, especially at high cardiac frequencies [14].

### Increased Diastolic SR Ca^2+^ Leak

The observation of increased diastolic SR Ca^2+^ leak in atrial myocytes is interesting since this is the first report showing that low aerobic capacity leads to a cellular substrate that may be more prone to triggering of atrial arrhythmias. Several studies on ventricle cardiomyocytes [22]–[24] and also from patients with atrial fibrillation [25] have shown that increased RyR2 Ca^2+^ leak from the SR during diastole is a potent trigger for uncontrolled electrical activity that may cause spontaneous contractions and arrhythmias. On this basis several novel Ca^2+^ release RyR2-stabilizing drugs have been proposed [26]. Phosphorylation of serine −2814 at the RyR2 by CaMKII is a well-documented cause of increased Ca^2+^ leak [17], [22], [27]. Although further studies including higher number of animals are necessary to elucidate the mechanism involved in the regulation of Ca^2+^ leak, our data indicates that RyR2 serine-2814 phosphorylation is apparently increased in the LCR rats. Importantly, this suggests a deleterious signaling induced by contrasting for low aerobic capacity.

## Conclusions

This study report for the first time that contrasting for low or high aerobic capacity leads to diverse functional and structural remodeling of atrial myocytes. Compared to rats with high aerobic capacity we found that low aerobic capacity in LCR rats was associated with reduced atrial myocyte contractility and diastolic relaxation that were associated with impaired Ca^2+^-handling. Reduced systolic Ca^2+^ amplitude in LCR rats was associated with reduced ability to initiate Ca^2+^ release from the SR that probably is caused by a less developed T-tubule network. Furthermore, low aerobic capacity in LCR rats led to an increased diastolic SR Ca^2+^ leak over the RyR2, which has been linked to cardiac arrhythmias in several studies on left ventricular myocytes. Our study therefore suggests that low aerobic capacity may lead to negative signaling in atrial myocytes with defective properties of Ca^2+^ handling that is not only negative for atrial function but also may cause a cellular substrate that is more prone for triggering of atrial arrhythmias. It is likely that the improved cardiomyocyte function and Ca^2+^ handling associated with high aerobic capacity has a positive effect during increased workload of the atria. It is furthermore tempting to speculate that the positive adaptations in the atrial cellular mechanisms may protect against atrial dysfunction such as atrial fibrillation.
